# Christmas at "The Foundling"

**Published:** 1887-01-08

**Authors:** S. K. McBride

**Affiliations:** Sister in charge of the Infirmary


### CHRISTMAS AT "THE FOUNDLING."

It may be of some interest to your readers to hear something of our doings in the Infirmary of the Foundling. I think my little patients had a right merry Christmas, all able to be up. They were entertained in the girls' ward, which we did our best to brighten up with decorations and toys. Twenty-two of us sat down to dessert at five o'clock, and a merry feast we had, what with crackers, and cakes, and other things too numerous to mention. Kind Mr. G—— came up and gave each child a silver coin, bright and fresh from the Mint, and Mrs. G—— gave each one a toy. There were drums for some, dolls for others, games for the bigger ones, and tambourines. Then we had games, and rides on the rocking-horse, until all, tired with play, went to bed, hugging toys, cards, etc. So we leave them to dream of Santa Claus, who will ever remain a stranger, yet always the friend of the little ones.

S. K. McBride,
Sister in charge of the Infirmary.

